# Nonthyroidal illness syndrome in acute pancreatitis patients: an 8-year cohort study

**DOI:** 10.1186/s12876-022-02111-5

**Published:** 2022-02-03

**Authors:** Cheng Qu, Zehua Duan, Xiaojia Xiao, Mei Wei, Kun Gao, Xianqiang Yu, Lu Ke, Zhihui Tong, Weiqin Li

**Affiliations:** 1 Center of Severe Acute Pancreatitis (CSAP), Department of General Surgery, Jinling Hospital, Medical School of Nanjing University, No. 305 Zhongshan East Road, Nanjing, 210002 Jiangsu Province China; 2 Center of Severe Acute Pancreatitis (CSAP), Department of General Surgery, Jinling Clinical Medical College of Southern Medical University, Nanjing, 210002 China; 3 Center of Severe Acute Pancreatitis (CSAP), Department of General Surgery, Jinling Clinical Medical College of Nanjing Medical University, Nanjing, 210002 China; 4 Center of Severe Acute Pancreatitis (CSAP), Department of General Surgery, Jinling Clinical Medical College of Southeast University, Nanjing, 210002 China

**Keywords:** Nonthyroidal illness syndrome, Pancreatitis, Thyroid hormone

## Abstract

**Background:**

Nonthyroidal illness syndrome (NTIS) is common in critical illness and is associated with poor prognosis. The aim of this study was to find the prevalence, charateristics, and prognosis of NTIS and its correlation with outcomes in AP patients.

**Methods:**

A retrospective review of AP patients with a diagnosis of NTIS from Jan 2012 to September 2020 was performed. The serum thyroidal hormone (TH) disturbances, as well as the demographic characteristics and clinical outcomes of the study patients, were collected and analyzed.

**Results:**

Over the eight years, 183 included AP patients were diagnosed as NTIS, constituting an incidence of 64.7%. Patients with NTIS were admitted with worse condition based on the higher APACHE II score, SOFA score, Balthazar's CT score, CRP and lower albumin than euthyroid patients. Also, these patients had a longer ICU duration (3, 2–10 vs 2, 0–3, days, *P* = 0.039) and tended to be more likely to develop infected pancreatic necrosis (IPN) (15.3% vs 6.3%, *P* = 0.087) and gastrointestinal fistula (6% vs 0%, *P* = 0.082) than euthyroid patients. Free triiodothyronine (FT3) was found the best performance in predicting death compared by other well-recognized biomarkers.

**Conclusion:**

NTIS is common in AP patients within 7 days after the onset of the disease. NTIS is associated with the worse characteristics at admission and poor outcome during the course. FT3 should be investigate as a potential biomarker in the prediction of death in AP patients.

## Background

Nonthyroidal illness syndrome (NTIS), also known as the low T3 syndrome or euthyroid sick syndrome, is characterized by low serum levels of free and total triiodothyronine (T3) with normal or low thyroid-stimulating hormone (TSH) [1, 2]. Such typical changes of the serum thyroidal hormone (TH) differ from those in primary or secondary thyroid disorders and refer to distortions in thyroid function without thyroid disease [3]. This syndrome is not uncommon and associated with adverse outcomes in many diseases, including infectious diseases [4], cardiovascular [5, 6], gastrointestinal diseases [7], trauma [8] and unselected critical illness patients [9–11].

Acute pancreatitis (AP) is an inflammatory disorder of the pancreas, consequently following by systemic inflammatory response [12]. Previous studies reported that moderately severe AP (MSAP) and severe AP (SAP) patients were tested with low FT3 levels than moderately AP (MAP) patients and monitoring free triiodothyronine (FT3) levels in the early stage of AP is helpful to evaluate disease severity [13, 14]. However, no studies have systematically analyzed the TH disturbances, the incidence of NTIS and the association between NITS and the clinical outcome in adult AP patients.

The present study was performed to evaluate the thyroid function and the prevalence, underlying mechanisms, and its correlation with clinical variables and prognosis of NTIS in adult patients with AP.

## Method

### Study design and participants

This study retrospectively screened all AP cases admitted to the Center of Severe Acute Pancreatitis, Department of General Surgery, Jinling Hospital, within 7 days from the onset of the disease, from Jan 2012 to September 2020. The diagnosis and classification of the severity of AP were according to the 2012 revision of the Atlanta Classification [15], based on at least two of the following three criteria: (i) abdominal pain suggesting AP, (ii) elevated serum amylase and/or lipase > 3 times the upper limit of normal, and (iii) characteristic AP computed tomography (CT) findings.

The exclusion criteria were as follows: (1) age less than 18 years or older than 70 years; (2) history of thyroidal, hypophyseal, or hypothalamic disease; (3) in pregnancy, with malignancy or autoimmune diseases, etc.; (4) medication history of thyroidal hormone or antithyroid drugs.

### Data collection

All the data were extracted from an electronic database (APDatabase) which were collected prospectively. This study was approved by the institutional review board (No: 2020 JLAPDMC-006). Routine written informed consent was obtained for data collection, storage, and academic use of data from all patients or next of kin at admission. Additional informed consent from individuals was waived due to the retrospective and anonymous nature of the current study.

The demographic data, diagnosis, imaging data, and management as well as clinical outcomes of the study subjects were collected. Levels of serum thyroidal hormone, including free triiodothyronine (FT3), total triiodothyronine (TT3), free thyroxin (FT4), total thyroxin (TT4), and thyroid-stimulating hormone (TSH) were investigated using the chemiluminescent microparticle immunoassay within the 24 h after admission. Other laboratory test results, including hemoglobin, hematocrit, platelet, C-reactive protein (CRP), IL-6, creatinine, and alanine aminotransferase (ALT) were obtained from the Central Laboratory of Jinling Hospital. Meanwhile, acute physiology and chronic health evaluation II (APACHE II) score and sequential organ failure assessment (SOFA) scores were manually calculated for each single patient.

### Diagnostic criteria of NITS

Nonthyroidal illness syndrome was defined as a low serum FT3 level (< 3.1 pmol/L) without an elevated TSH level (< 0.3 mIU/L). The normal ranges of serum thyroidal hormone and thyroid-stimulating hormone in our hospital were as follows: FT3, 3.1–6.5 pmol/L; TT3, 1.23–3.07 nmol/L; FT4, 7.9–17.2 pmol/L; TT4, 71–161 nmol/L; and TSH, 0.3–4.5 mIU/L. According to symptoms and thyroid function assays tested within 24 h after admission, patients were categorized into five groups: euthyroid, NTIS, subclinical hypothyroidism, subclinical hyperthyroidism, and hypothyroidism.

### Statistical analysis

Data involving demographics, AP etiologies, underlying diseases, smoking and drinking, clinical outcomes and management were compared between patients in the euthyroid and the NITS groups. The categorical variables (sex, etiology, underlying disease, smoking, drinking, clinical outcome, et al.) were described using frequency and percentage. Continuous variables (age, BMI, severity score, laboratory tests, TH and TSH levels, et al.) were described using mean values ± SD or median with interquartile range (IQR). We used the t-test to compare the continuous variables of the normal distribution, and the Mann–Whitney U tests compare the nonnormally distributed variables. For categorical variables, a chi-square test or Fisher exact test was used. Statistical significance was set at two-sided *P* < 0.05. Area Under the Curve of the Receiving Operating Characteristic Curve (AUCROC) analysis was used to define the optimal cutoff point of some important factors to predict the death in AP patients. All data were analyzed using SPSS 19.0 statistical software (SPSS Inc., Chicago, IL).

## Results

**Fig. 1:**
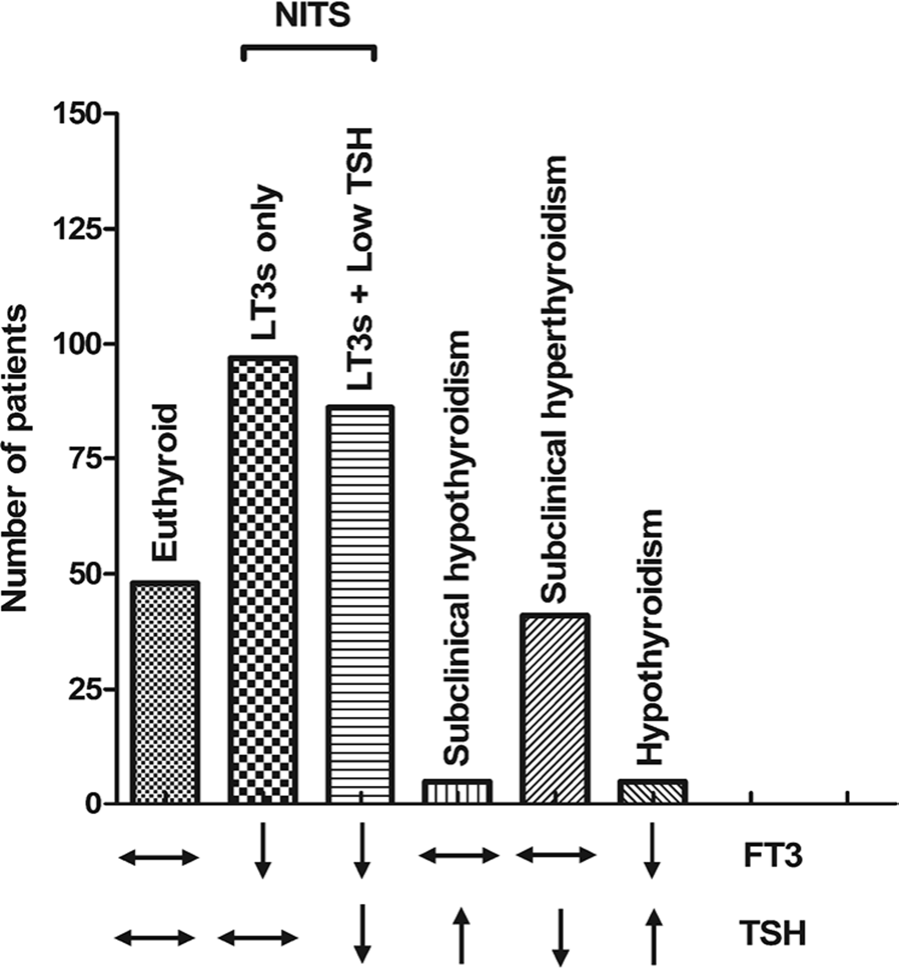
Distribution of thyroidal laboratory values in the included patients (n = 283). *NITS* nonthyroidal illness syndrome, *FT3* free triiodothyronine, *TSH* thyroid-stimulating hormone, *LT3s* low-T3 syndrome

In our study, a total of 283 patients were included eventually. As shown in Fig. 1, the included patients were divided into five groups according to the thyroidal characteristics of each group listed at the bottom. Among them, 48 (48/283, 17.0%) patients were euthyroid with normal FT3, FT4 and TSH levels. One hundred and eighty-three patients (183/283, 64.7%) were diagnosed as NITS with a low serum FT3 level without elevated TSH level. NITS patients were subdivided into two groups: low FT3 only group (97/183, 43.0%) and low FT3 with concomitant low TSH group (86/183, 47.0%). Five patients (5/283, 1.8%) were classified as subclinical hypothyroidism, 41 patients (41/283, 14.5%) as subclinical hyperthyroidism and 5 patients (5/283, 1.8%) as subclinical hyperthyroidism based on the serum FT3 and TSH level.

### Baseline characteristics

Demographics and baseline characteristics of patients between the euthyroid group and the NITS group are listed in Table 1. Patients in the NTIS group were admitted with higher APACHE II score (6.9 ± 5.0 vs 4.1 ± 3.5, *P* < 0.001), SOFA score (2.5 ± 3.0 vs 1.2 ± 1.9, *P* < 0.001), CTSI score (6.3 ± 2.0 vs 4.9 ± 1.7, *P* < 0.001), CRP (154.1 ± 79.9 vs 127.0 ± 81.3, mg/L, *P* = 0.039) and lower albumin (30.9 ± 4.3 vs 33.0 ± 5.8, g/L, *P* = 0.004) with a significant difference. And the Patients in the NITS had less percentage of hypertension (33/183, 18.0% vs 16/48, 33.3%, *P* = 0.021). However, there was no difference in demographics (age, sex and BMI) and the distribution of AP etiology and diabetes mellitus (DM).

**Table 1:** Baseline characteristics and clinical variables between Euthyroid and NTIS groups

Variables	All N = 231	Euthyroid Group N = 48	NITS Group N = 183	*P* value
Age, year, median (IQR)	47 (38.0–55.0)	48.5 (37.5–54.0)	47.0 (38.0–55.0)	0.666
Sex, M/F	173/92	36/12	114/69	0.101
BMI, kg/m^2^, median (IQR)	26.5 (24.1–28.3)	26.2 (24.3–28.1)	26.7 (23.3–28.6)	0.432
Etiology, n (%)				
Biliary	96 (41.6)	16 (33.3)	80 (43.7)	0.194
Alcohol	10 (4.3)	2 (4.2)	8(4.4)	0.950
Hyperlipidemia	119 (51.5)	28 (58.3)	91 (49.7)	0.288
Others	8 (3.5)	2 (4.2)	6 (3.3)	0.126
Underlying diseases, n (%)				
Hypertension	49 (21.2)	16 (33.3)	33 (18.0)	0.021
DM	44 (19.0)	9 (18.8)	35 (19.1)	0.953
Smoking, n (%)		14 (29.2)	45 (24.6)	0.518
Drinking, n (%)		13 (27.1)	42 (23.0)	0.550
APACHE II score, median (IQR)	6.4 ± 4.9	4.1 ± 3.5	6.9 ± 5.0	< 0.001
SOFA score at admission, median (rIQR)	2.2 ± 2.9	1.2 ± 1.9	2.5 ± 3.0	< 0.001
Balthazar’s CT score, median (IQR)	6.0 ± 2.0	4.9 ± 1.7	6.3 ± 2.0	< 0.001
CRP, mg/L, mean ± SD	148.5 ± 80.6	127.0 ± 81.3	154.1 ± 79.9	0.039
WBC, mean ± SD	11.5 ± 4.7	10.8 ± 4.6	11.7 ± 4.8	0.260
Hemoglobin, mean ± SD	110.5 ± 22.4	114.6 ± 22.6	109.4 ± 22.3	0.155
Albumin, g/L, mean ± SD	31.3 ± 4.7	33.0 ± 5.8	30.9 ± 4.3	0.004

*BMI* body mass index; *IQR* interquartile range; *DM* diabetes mellitus; *APACHE II* acute physiology and chronic health evaluation II; *SOFA* sequential organ failure assessment; *CRP* C-reactive protein; *WBC* white blood cell

The level of serum thyroid hormone and thyroid-stimulating hormone are presented in the Table 2. The FT3 (2.5, 2.2–2.8, vs 3.4, 3.3–4.3, *P* < 0.001), FT4 (9.0, 7.8–10.5 vs 10.6, 9.3–11.5, *P* < 0.001), TT3 (0.4, 0.3–0.5 vs 0.6, 0.3–0.6, *P* < 0.001), TT4 (59.0, 45.7–74.5 vs 75.3, 62.3–90.6, *P* < 0.001) and TSH (0.5, 0.3–0.9 vs 0.9, 0.6–1.7, *P* < 0.001) were significantly lower in the NITS group than in the euthyroid group. The level of thyroglobulin (TG) (4.4, 2.5–8.2 vs 6.1, 3.2–8.7, *P* = 0.404) and parathyroid hormone (PTH) (4.6, 2.6–9.1 vs 3.6, 2.3–7.2, *P* = 0.855) showed no significant differences.

**Table 2:** Thyroid hormones parameters between Euthyroid and NTIS groups

Variables	All	Euthyroid Group	NITS Group	*P* value
FT3 (pmol/L)	2.6 (2.3–3.0)	3.4 (3.3–4.3)	2.5 (2.2–2.8)	< 0.001
FT4 (pmol/L)	9.4 (8.2–10.9)	10.6 (9.3–11.5)	9.0 (7.8–10.5)	< 0.001
TT3 (nmol/L)	0.4 (0.3–0.6)	0.6 (0.4–0.8)	0.4 (0.3–0.5)	< 0.001
TT4 (nmol/L)	62.5 (48.1–80.4)	75.3 (62.3–90.6)	59.0 (45.7–74.5)	< 0.001
TSH (mU/L)	0.6 (0.4–1.1)	0.9 (0.6–1.7)	0.5 (0.3–0.9)	< 0.001
TG (μg/L)	4.8 (2.8–8.2)	6.1 (3.2–8.7)	4.4 (2.5–8.2)	0.404
PTH (pmol/L)	4.4 (2.5–7.9)	3.6 (2.3–7.2)	4.6 (2.6–9.1)	0.855

*FT3* free triiodothyronine; *TT3* total triiodothyronine; *FT4* free thyroxin; *TT4* total thyroxin; *TSH* thyroid-stimulating hormone; *TG* thyroglobulin; *PTH* parathyroid hormone

### Clinical outcomes

**Fig. 2:**
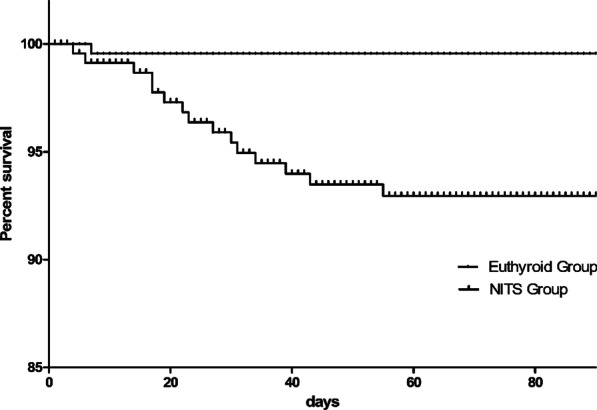
The cumulative survival rates in the euthyroid and NTIS groups. *NITS* nonthyroidal illness syndrome

The clinical outcomes of included patients are shown in Table 3. Acute pancreatitis patients (≤ 7 days from the onset of abdominal pain) with NITS diagnosed within the 24 h after admission had a longer ICU duration than the euthyroid group (3, 2–10 vs 2, 0–3, *P* = 0.039) significantly and tended to have higher mortality but not significantly (18, 9.8% vs 1, 2.1%, *P* = 0.082). Figure 2 compares the cumulative survival rates in the euthyroid and NTIS groups. Also, more percentage of AP patients with NITS developed infected pancreatic necrosis (IPN) (29, 15.3% vs 3, 6.3%, *P* = 0.087) and gastrointestinal fistula (11, 6% vs 0, 0%, *P* = 0.082) without significant difference compared with euthyroid patients. Along, the need for percutaneous catheter drainage (PCD) for pancreatic necrosis, which is the first step of a step-up method for pancreatic necrosis infection, was significantly more in the NITS group than the euthyroid group. What’s more, the requirement of open pancreatic necrosectomy tended to be higher in the NITS group. However, the percentage of other complications had no significant difference, including intra-abdominal hemorrhage, new onset of organ failure and multiple organ dysfunction syndromes (MODS).

**Table 3:** Clinical outcomes between Euthyroid and NTIS groups

Outcomes	All N = 231	Euthyroid Group N = 48	NITS Group N = 183	*P* value
Hospital mortality, n (%)	19 (8.2)	1 (2.1)	18 (9.8)	0.082
ICU duration, median (IQR), d	3 (2–8)	2 (0–3)	3 (2–10)	0.039
Intra-abdominal hemorrhage, n (%)	14 (6.1)	1 (2.1)	13 (7.1)	0.194
Gastrointestinal fistula, n (%)	11 (4.8)	0 (0)	11 (6.0)	0.082
New onset OF, n (%)				
Respiratory	34 (14.7)	7 (14.6)	27 (14.8)	0.976
Renal	17 (7.4)	5 (10.4)	12 (6.6)	0.362
Cardiovascular	7 (3.0)	1 (2.1)	6 (3.3)	0.667
MODS	35 (15.2)	5 (10.4)	30 (19.0)	0.304
IPN, n (%)	31 (13.4)	3 (6.3)	29 (15.3)	0.087
PCD, n (%)	34 (14.7)	2 (4.2)	32 (17.5)	0.020
Open pancreatic necrosectomy, n (%)	10 (4.3)	0 (0)	10 (5.5)	0.098

*IQR* interquartile range; *ICU* intensive care unit; *OF* organ failure; *MODS* multiple organ dysfunction syndromes; *IPN* infected pancreatic necrosis; *PCD* percutaneous catheter drainage

### Evaluation of the prognostic value of the candidate predictors of death in AP patients

**Fig. 3:**
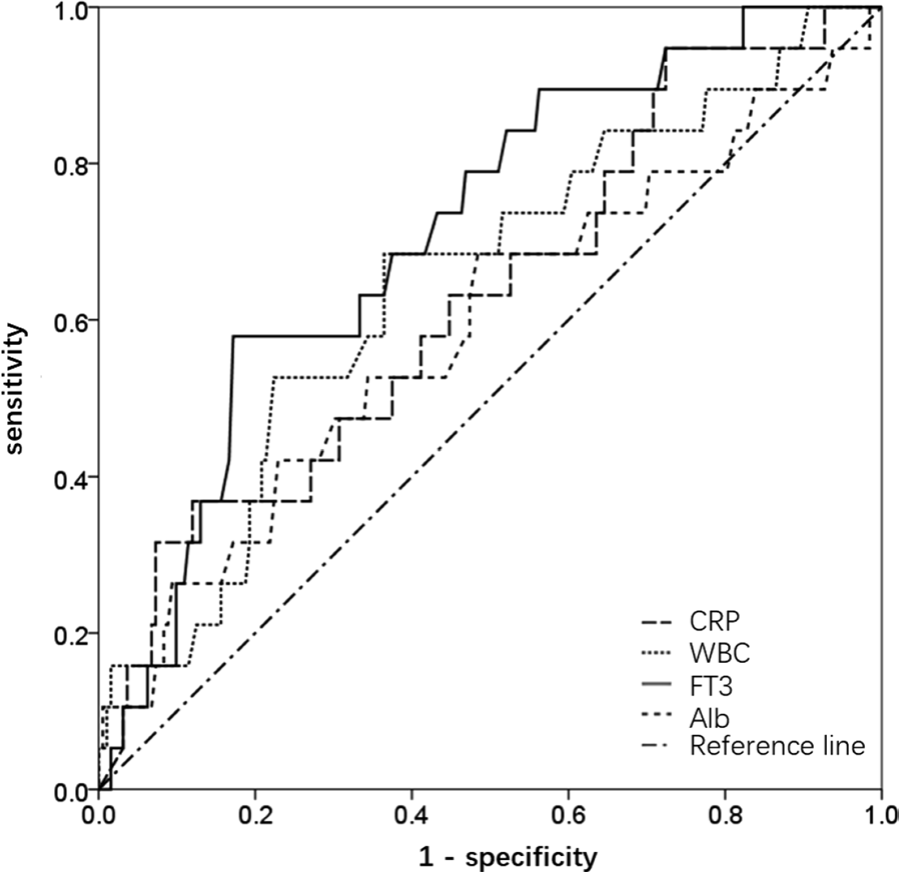
The ROC analysis of FT3, CRP, WBC and Alb in the prediction of death in AP patients. *ROC* receiving operating characteristics, *FT3* free triiodothyronine, *CRP* C-reactive protein, *WBC* white blood cell, *Alb* albumin

The AUCROC for FT3 was 0.714 (95% CI 0.602–0.827) in predicting death in AP patients (Fig. 3). ROC analysis revealed that the serum FT3 cutoff point of 2.2 pmol/L had optimal predictive value for the death in AP patients. We compared the FT3 with other common serum laboratory tests, including WBC, CRP and Alb in predicting the death of AP patients by ROC analysis, showing that FT3 was of the best performance.

## Discussion

To our knowledge, the present 8-year population-based retrospective study is the largest clinical investigation of the TH disturbances (< 7 days after the onset of disease) and the first study to explore the role of NTIS in AP patients. Furthermore, we identified FT3 as a valuable predictor of the death of AP patients among the TH, which showed the best predictive performance compared with routine biomarkers by the ROC analysis.

NITS are present in more than half of included AP patients with a prevalence of 64.7%. There is some possible pathogenesis: (1) in the acute phase of AP, patients are in a poor nutritional status treated with fasting, which induces a decrease in serum TH through a multifactorial mechanism including a decrease in serum leptin, and downregulation of hypothalamus-hypophysis-thyroid axis, resulting in persistently low serum TSH level [16]. (2) activity of hepatic thyroxine 5’-deiodinase (deiodinases type 1, D1) is impaired in AP patients by hepatic injury through endotoxins, cytokines released by the inflammatory pancreas, etc., sequentially affecting the conversion from T4 to T3 [17–19]. (3) the reduced the levels of thyroid hormone-binding protein and its impaired binding activity, thereby increase the clearance of the free hormone [20].

NTIS is commonly considered as an adaptive mechanism to the overall downregulate of metabolism in the organism to save energy in critically ill patients [21]. On the one hand, the important FT3 target organs, such as the liver and muscles, are affected by the decrease thyroid hormone, of which the downregulation metabolism might be beneficial in these patients. On the other hand, the critically ill patients with NTIS may develop some complications due to the low active organ, including intestine, cardiovascular, kidney, etc. [3, 22, 23].

For the clinical characteristics at admission, we discovered higher APACHE II, SOFA and CTSI scores, a higher serum CRP level and a lower serum albumin level in the NTIS group, which is consistent with the clinical characteristics of these patients reported previously [9, 22]. They were probably exposed to worse clinical outcomes and prognosis.

For the clinical outcome in these patients, the percent of developing IPN, along with the need for invasive intervention, including PCD and open pancreatic necrosectomy is higher in the NTIS group. IPN causes high overall mortality of around 5 percent in acute pancreatitis [24–26] As reported previously, serum FT3 can directly modulate immune responses, including increasing phagocytic activity of immune cells, lymphocyte proliferation, antibody production, cytokine production, cytokine receptor expression, and oxygen-free radical generation through both genomic and nongenomic mechanisms [27]. Further, iodothyronines may have the effect of local antimicrobial by releasing liberated iodide ions at the site of pancreatic necrosis [2]. What’ more, the longer ICU duration is found in the NTIS group than the euthyroid group, which reflected the slower recovery in NTIS due to the downregulated metabolism. So, Monitoring the TH level can help identify the population of the high risk of developing IPN in AP patients and predict the course of the disease.

Previous studies reported that FT3 was a valuable and feasible biomarker to evaluate and predict the classification of the AP severity [13, 14]. We found that FT3 is of the better predictive performance compared with other well-recognized clinical variables, including WBC, CRP and Alb by ROC analysis. WBC and CRP are inflammatory markers that are reported to determine the severity of AP [28]. Alb is exclusively synthesized in the liver and low serum Alb (< 35 g/L) was found independently associated with an increased risk of developing of persistent organ failure and death in acute pancreatitis [29–31]. FT3 is a biomarker reflecting thyroid function that respond early in the AP course or serve as a self-regulating mechanism to changes in energy consumption [13]. FT3 may be useful, combined with other biomarkers, for the development of a therapeutic strategy and the ultimate improvement of the outcome.

There are certain limitations of the present study. Firstly, the low mortality in this study cohort makes the results of the ROC curves prone to bias. Moreover, this is a single-center retrospective study with limited sample size so that the association between NITS and clinical outcomes does not necessarily imply causality. For the treatment, whether interventions aimed at regulating TH concentrations in patients with AP are beneficial has not been satisfactorily answered yet. More studies should be taken to investigate the effect of normalizing TH concentration in AP patients.

## Conclusion

In conclusion, NTIS is common in adult patients with AP within 7 days after the onset of the disease. NTIS is associated with the worse characteristics at admission and poor prognosis during the course. FT3 is a potential biomarker in the prediction of death in AP patients.

## Data Availability

The datasets analysed during the current study are available from the corresponding author on reasonable request.

## Abbreviations

NITS: Nonthyroidal illness syndrome
T3: Triiodothyronine
TSH: Thyroid-stimulating hormone
TH: Thyroidal hormone
AP: Acute pancreatitis
MSAP: Moderately severe acute pancreatitis
SAP: Severe acute pancreatitis
MAP: Moderately acute pancreatitis
FT3: Free triiodothyronine
CT: Computed tomography
TT3: Total triiodothyronine
FT4: Free thyroxin
TT4: Total thyroxin
CRP: C-reactive protein
ALT: Alanine aminotransferase
APACHE II score: Acute physiology and chronic health evaluation II score
SOFA score: Sequential organ failure assessment score
BMI: Body mass index
SD: Standard deviation
AUCROC: Area under the curve of the receiving operating characteristic curve
DM: Diabetes mellitus
TG: Thyroglobulin
PTH: Parathyroid hormone
IPN: Infected pancreatic necrosis
PCD: Percutaneous catheter drainage
MODS: Multiple organ dysfunction syndromes
WBC: White blood cell
